# NSDHL-containing duplication at Xq28 in a male patient with autism spectrum disorder: a case report

**DOI:** 10.1186/s12881-018-0705-7

**Published:** 2018-10-30

**Authors:** Chun-Chun Hu, Yun-Jun Sun, Chun-xue Liu, Bing-rui Zhou, Chun-yang Li, Qiong Xu, Xiu Xu

**Affiliations:** 1 Department of Child Healthcare, Children’s Hospital of Fudan University, Shanghai, 201102 China; 2 Institute of Neuroscience, State Key Laboratory of Neuroscience, CAS Center for Excellence in Brain Science and Intelligence Technology, Chinese Academy of Sciences, Shanghai, 200031 China

**Keywords:** Xq28 duplication, *NSDHL*, Autism, CNV

## Abstract

**Background:**

Autism spectrum disorder (ASD) is a neurodevelopmental disorder in which genetics plays a key aetiological role. The gene encoding NAD(P)H steroid dehydrogenase-like protein (*NSDHL*) is expressed in developing cortical neurons and glia, and its mutation may result in intellectual disability or congenital hemidysplasia.

**Case presentation:**

An 8-year-old boy presented with a 260-kb NSDHL-containing duplication at Xq28 (151,868,909 – 152,129,300) inherited from his mother. His clinical features included defects in social communication and interaction, restricted interests, attention deficit, impulsive behaviour, minor facial anomalies and serum free fatty acid abnormality.

**Conclusion:**

This is the first report of an ASD patient with a related NSDHL-containing duplication at Xq28. Further studies and case reports are required for genetic research to demonstrate that duplication as well as mutation can cause neurodevelopmental diseases.

**Electronic supplementary material:**

The online version of this article (10.1186/s12881-018-0705-7) contains supplementary material, which is available to authorized users.

## Background

Autism spectrum disorder (ASD) is a neurodevelopmental disorder that is defined in DSM-5 [1] as persistent deficits in social communication and social interaction across multiple contexts in conjunction with restricted, repetitive patterns, interests, or activities as manifested by at least two prototypically inflexible behaviours [2]. A high degree of heritability and modest environmental influences contribute to the aetiology of ASD [3, 4]. The genetic architecture of autism has proven to be complex and heterogeneous through linkage or association based on whole-genome or exome sequencing [5, 6]. In recent decades, 10–20% of ASD cases have been attributed to ASD susceptibility genes [6]. In the X chromosome, double expression of these related genes leads to functional disomy [7–9]. Among the pathogenic X-linked duplications, the most prominent and well-studied is that of the Methyl-CpG-binding Protein 2 gene (*MECP2*; OMIM: 300005), located at Xq28, causing severe X-linked intellectual disability (XLID) [10], and its loss of function causes Rett syndrome (OMIM: 613454) [11, 12]. Duplication of chromosome 15q11–13, which includes a series of imprinting and non-imprinting genes, results in the recurrent cytogenetic abnormalities associated with ASD and represents one of the most frequently reported CNVs in ASD [13].

Duplication involving the int22h-1/int22h-2 LCR-flanked region in distal Xq28 was recently linked to intellectual and developmental disabilities [14, 15]. NAD(P)H steroid dehydrogenase-like protein (*NSDHL*), also located at Xq28, is expressed in developing cortical neurons and glia and encodes an enzyme in post-squalene cholesterol biosynthesis [16]. Mutations of *NSDHL* have been related to neurodevelopmental diseases including CHILD syndrome (OMIM: 308050) and CK syndrome (OMIM: 300831). In CHILD syndrome, *NSDHL* function is presumably eliminated or greatly reduced [17]; CK syndrome affects only males [16]. CK syndrome caused by *NSDHL* mutations is characterized by mild to severe cognitive impairment, seizures beginning in infancy, microcephaly, cerebral cortical malformations and a thin body habitus [18]. Most male patients with CK syndrome manifest behaviours of aggression, attention deficit hyperactivity disorder (ADHD), and irritability. Using Autism Diagnostic Interview-Revised (ADI-R) [19] or the Autism Diagnostic Observation Schedule (ADOS) [20], the affected males fulfil partial criteria for ASD [18]. However, little is known about the clinical consequences of *NSDHL* duplication, and no *NSDHL* whole-gene duplication with ASD has been reported in the literature.

Here, we report an 8-year-old boy diagnosed with ASD and possessing a 260-kb *NSDHL*-containing duplication at Xq28 inherited from his mother; this constitutes the first report of an ASD patient with a related *NSDHL*-containing duplication at Xq28. The patient underwent careful clinical observation, detailed examination, and comprehensive medical history recording and demonstrated symptoms of social deficiency, restricted interests, attention deficit and impulsive behaviour but normal cognition.

## Case presentation

### Subject

**Fig. 1:**
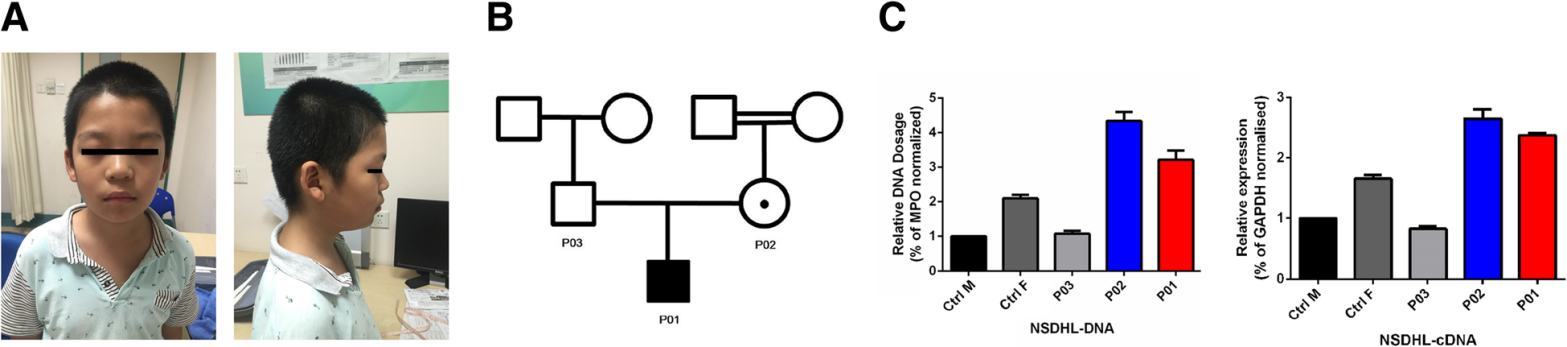
**a** Photographs of P01. P01 represents the patient. Minor facial anomalies include a depressed nasal bridge and thick, drooping upper eyelids. **b** Pedigree of family P01. Standard pedigree symbols are used. P01 represents the patient, P02 represents the mother of the patient, and P03 represents the father. **c** Confirmation of results using real-time qPCR. Ctrl M and Ctrl F are male and female controls with normal *NSDHL* gene dosage/expressions. P01 represents the patient, P02 represents the mother of the patient, and P03 represents the father of the patient. The crossing point (CP) values of each sample were normalized to that of a housekeeping gene, MPO/GAPDH. Error bars denote the standard deviation for five DNA/RNA samples. All data are shown as the mean ± SEM. P01 had inherited the *NSDHL* duplication from P02, and P03 carried a normal sequence

The patient was referred to our clinic because of indiscipline and impulsive behaviour. He was born at full-term after a normal pregnancy by caesarean section and had a birth weight of 2800 g. During the neonatal period, he had been hospitalized twice, once for pathological jaundice (at 2 months of age) and again for hernia (at 8 months of age). Otherwise, development was nearly normal. The patient walked unsupported at the age of 15 months but exhibited poor motor coordination and slight hypotonia. He had asthma at 3–4 years of age but gradually improved, although with continued allergic rhinitis. He seldom exhibited sharing behaviours or facial expressions and exhibited repetitive patterns of behaviour. He displayed poor linguistic competence, deficits in social reciprocity, and difficulty in maintaining normal back-and-forth conversation with normal eye contact. His ADOS-M3 [20] and ADI-R scores [19] were all above the cutoff (See Additional file 1: Table S1). In primary school, it was difficult for the subject to obey class discipline due to his impulsive behaviours and attention deficit. SNAP-IV [21] was administered to screen for ADHD (See Additional file 1: Table S1). According to the clinical data and assistant examination, the patient fulfilled the diagnostic criteria for ASD and ADHD. Physical examination revealed minor facial anomalies, including a depressed nasal bridge and thick, drooping upper eyelids (Fig. 1a).

At reexamination at the age of 9 years and 8 months, the patient remained weak in language comprehension and had a short attention span. His height was 146.5 cm, and his weight was 42.15 kg (within the 90 to 95 centile for his age). Based on the Wechsler Intelligence Scale for Children (WISC) [22], his IQS of Verbal Scale was 96, and his IQS of Performance Scales was 90, producing a Full Scale IQ 92 (See Additional file 1: Table S1). The results showed that he had normal cognition with a high score for calculation but a low score for comprehension. After examination of the serum lipids, he showed abnormal serum free fatty acids (875 μmol/L (age 9) and 1165 μmol/L (age 10)); the normal levels were 172–588 μmol/L in age-matched healthy children. No significant abnormalities were detected via electroencephalogram (EEG) or head magnetic resonance imaging (MRI).

Both of the patient’s parents completed the combined Raven’s Test (CRT) [23] and scored in the normal range. When consulting with both parents, we observed that the mother, who carried the *NSDHL*-containing duplication, exhibited a quick temper and irritability. Tracing the history of the pregnancy and delivery indicated that she may have suffered from postpartum depression. Although she did not have ASD symptoms, she did exhibit some of the phenotype. Her score for the Broader Autism Phenotype Questionnaire was high-normal. She reported no health issues or miscarriages. The father of the patient had a moderate temperament and was otherwise normal.

The gathered family history showed that the maternal grandparents of the patient had intermarried (they are cousins). DNA samples could not be obtained from either grandparent. Both parents of the patient were the only child in their respective families. Both families declared no significant health or psychological issues. (Fig. 1b).

Genomic DNA and RNA were extracted from the peripheral blood of the patient and his parents. The Agilent array-based comparative genomic hybridization (aCGH) microarray was employed to perform a genome-wide CNV scan of the patient using SurePrint G3 Human CGH microarrays 4 × 180 K (Agilent Technologies) following the manufacturer’s instructions. For WES of the family trio, the SureSelect Human All Exon V5 Kit (Agilent), the Illumina Hiseq X™ HD PE Cluster and SBS kit were used for exon capture, and the Illumina HiSeq X10 system was for paired-end parallel sequencing. To verify the source of *NSDHL* duplication in this patient and his parents, a primer set was designed to target the fragment along the gene through an online Primer Quest Tool (https://pga.mgh.harvard.edu/primerbank/) (See Additional file 1: Table S2). The presence of the duplication was also examined in the patient’s parents. X-chromosome inactivation patterns (XCIP) of the mother’s genomic DNA were tested by determining differential CpG-methylation. Genomic DNA/RNA samples from normal male and female individuals were used simultaneously as controls. Subsequent reverse transcription of RNA to cDNA was carried out using the Takara cDNA Synthesis Kit. qPCR was carried out in triplicate using the SuperReal PreMix Plus (SYBR Green) PCR reagent kit according to the manufacturer’s protocol.

**Fig. 2:**
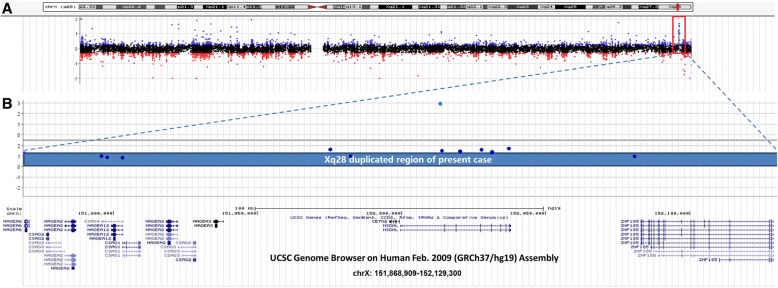
**a** The chromosomal status of the patient. Dots represent relative intensities reported in log2 ratios and the genomic locations of the oligonucleotide probes employed in the aCGH assay. A value of zero indicates a balanced chromosomal status. For the patient (P01), red (loss), black (no change), and blue (gain) dots represent log2 ratio deviations from the horizontal line of 0. Regions with copy number gains are indicated with red frames. **b** *NSDHL*-containing 260 kb Xq duplication in the patient. The blue bar indicates the Xq28 duplication region of the patient (chrX:151,868,909-152,129,300, converted to GRCh37/hg19) displayed using the UCSC genome browser. Blue (gain) dots represent log2 ratio deviations from the horizontal line of 0, and genes in the duplication region are indicated using the UCSC Genes prediction track

The patient fulfilled the criteria for ASD diagnosis. His clinical features included defects in social communication and interaction, restricted interests, attention deficit, impulsive behaviour, minor facial anomalies and showed abnormal serum free fatty acids. Array-CGH analysis identified a 260-kb duplication on chromosome Xq28 (151,868,909 – 152,129,300; GRCh 37/hg19) in the patient (Fig. 2a). The duplicated region (Fig. 2b), starting from MAGE2 to a partial copy of ZNF18, included the following genes: melanoma antigen (*MAGE*) family (*MAGE2, MAGEA3, MAGEA6, MAGEA12*), chondrosarcoma-associated gene (*CSAG1, CSAG2, CSAG3, CSAG4*), centrin (*CETN2*), NAD(P)H steroid dehydrogenase-like protein (*NSDHL*), and a part of zinc finger protein 185 (*ZNF18*). The qPCR result of DNA confirmed that the patient had inherited the *NSDHL* duplication from his mother and that his father carried a normal sequence; RNA analysis showed that the patient expressed almost threefold the RNA compared to normal males (Fig. 1c). We tested the X-chromosome inactivation patterns (XCIP) of the mother by determining the differential CpG-methylation. According to the pyrosequencing results, there was no significantly skewed X inactivation in the mother (See Additional file 2). The WES of the core family trio did not reveal any causative variants (See Additional file 3).

## Discussion and conclusions

The patient in our study manifested core symptoms of ASD with accompanying ADHD and impulsiveness. CNV scanning revealed that the patient had a Xq28 duplication encompassing 8 genes, and that the most disease-associating gene *NSDHL* was among this duplication region; this is the first report of an ASD patient with a related *NSDHL*-containing duplication at Xq28.

We hypothesized that the *NSDHL*-containing duplication at Xq28 was responsible for the ASD. Four *MAGE* family genes [24] have been reported to be expressed at high levels in numerous tumours of various histological types, whereas in a large panel of healthy tissues, expression was observed only in the testis and placenta. *CSAG* family genes [25], which are expressed at quite low levels in brain, are mostly expressed in the testis, and *CSAG1* is expressed in some higher-grade chondrosarcomas, chondrosarcoma-derived cell lines, and melanoma cell lines, as well as in some types of cartilage. *CETN2* [26] is associated with the centrosome, whereas *ZNF185* [27] is mostly expressed in the human adult prostate, testis, ovary, placenta, peripheral blood, and embryonic kidney; a partial copy was contained in this region (encompassing a minimum of 16 and a maximum of 18 exons). Since the levels of *CETN2* and *ZNF185* are also high in the brain, we searched more databases, including OMIM, Gene Cards and ClinVar, but still did not find any report indicating associations among *CETN2*, *ZNF185* and neural development disorders. Diseases associated with *CETN2* include Xeroderma pigmentosum [28], while diseases associated with *NSDHL* include CK Syndrome and CHILD Syndrome. Thus, most genes in the duplicated region are expressed beyond the brain or exhibit little relation to autism or neurodevelopmental disease (See Additional file 1: Table S3–1, S3–2). *NSDHL*, which is located at Xq28, is expressed in developing cortical neurons and glia and encodes an enzyme that functions in post-squalene cholesterol biosynthesis [16], thus affecting the steps of the cholesterol biosynthetic pathway. Research has shown that perturbations of cholesterol metabolism are associated with a variety of CNS disorders found in ASD [29] and other mental disorders [30]. Nevertheless, we still need more evidence to evaluate the effects of other genes in the duplicated region and more functional studies to show the importance of *NSDHL* to the development of ASD.

*NSDHL* mutations, which have been implicated in CK syndrome [18] in males, result in rare X-linked intellectual disabilities and cognitive impairment. Affected males also have behavioural problems, including aggression, ADHD, irritability, and seizures [18] and partially fulfil the criteria for ASD [18]. Our patient exhibited the characteristics of attention deficit and irritability but not seizure and satisfied the criteria for ASD diagnosis. The patient had an *NSDHL* duplication at Xq28, which was inherited from his mother. *NSDHL* is likely the most important disease-related gene within the duplicated region of this patient because the other genes in this duplicated region have little relevance to ASD. In contrast to patients with CK syndrome [16], our patient exhibited normal cognition, possibly because the *NSDHL* gene was duplicated and not mutated. His mother, who carried the *NSDHL* duplication on only one X chromosome, exhibited a few symptoms of ASD and had normal physical features. Her intellect and irritable disposition were consistent with previous reports of female carriers of *NSDHL* mutations [16]. Studies have demonstrated that female carriers of an *NSDHL* mutation generally do not manifest signs or symptoms of CK syndrome [31]. These carriers have normal physical features, intellect, and brain imaging findings but exhibit behavioural problems, such as irritability and aggression.

We also checked the common variation in DGV (http://dgv.tcag.ca/dgv/app/home) for the same CNV duplication region. We found that Jakobsson’s [32] study showed an observed gain including the entire *NSDHL* gene. Tracing the duplication region, we found out that a female had the duplication by checking the information of the raw data (https://www.ncbi.nlm.nih.gov/geo/query/acc.cgi?acc=GSM264524; sample ID HGDP00959). We assumed that female carriers with *NSDHL* duplication exhibit few symptoms and tend to be normal. To our knowledge, the frequency of the CNV in the normal population remains uncertain.

Patients with similar duplications reported in the DECIPHER database share some phenotypes and exhibit intellectual disabilities, muscular hypotonia and obesity. Table 1 shows an overview of similar duplications in DECIPHER. We found one boy (No. 258546) who had inherited from his unaffected mother a similar duplication region as that in our patient also had Autism Spectrum Disorder (We contacted the clinician for more information on these patients via DECIPHER. However, some patients did not have any further follow-up. A negative finding for ASD diagnosis in DECIPHER does not necessarily indicate that the cases do not have ASD; therefore, the importance of *NSDHL* cannot be ignored). Furthermore, because *NSDHL* functions in the removal of two C-4 methyl groups in one of the later steps of cholesterol biosynthesis, it is more likely that patients with similar duplications will exhibit obesity, including our patient, who showed higher serum levels of free fatty acids. These data support the dysfunction of *NSDHL* in cholesterol biosynthesis and the development of CNS.

**Table 1:** Clinical features of patients with similar duplications reported in the DECIPHER database^a^

Patient NO	Sex	Chromosome	Size	Inheritance	Gene number	Phenotype
258546	46XY	X	73.22 kb	From an unaffected mother	2	Autism Spectrum Disorder
		151958561-152031784				
268469	46XY	8	273.31 kb	From a normal parent	1	Obesity Low anterior hairline Intellectual disability
		13745710-14028750				
		X	283.04 kb	From a normal parent	11	
		151897058-152170367				
251730	46XX	X	1.33 Mb	De novo	45	Bulbous nose Short palm Muscular hypotonia
		151901372-153232466				
288269	46XX	X	63.38 kb	Unknown	1	Global developmental delay
		122322494-1223858575				
		X	609.21 kb	Unknown	18	
		151492204-152101413				
290161	46XY	1	89.38 kb	Unknown	1	Hypoplastic male external genitalia Obesity
		225217624-225307001				
		16	609.48 kb	Unknown	32	
		29586128-30195608				
		X	160.02 kb	Unknown	3	
		151958995-152119014				
249396	46XY	X	11.55 Mb	De novo	122	Low-set ears Hypertelorism Cryptorchidism Micrognathia Muscular hypotonia Intellectual disability Prenatal short stature
		140664743-152216545				

^a^This study makes use of data generated by the DECIPHER [34] community. A full list of centres who contributed to the generation of the data is available from http://decipher.sanger.ac.uk and via email from decipher@sanger.ac.uk. Funding for the project was provided by the Wellcome Trust. DECIPHER: Database of Chromosomal Imbalance and Phenotype in Humans using Ensembl Resources. Firth, H.V. et al. (2009). Am.J.Hum.Genet 84, 524–533 (Doi:dx.doi.org/10/1016/j.ajhg.2009.03.010)

Here, we have identified an 8-year-old patient carrying a 260-kb *NSDHL*-containing duplication inherited from his mother. Since the data in DGV and DECIPHER are not considered part of an peer-reviewed publication, this is the first report of an ASD patient with a related *NSDHL*-containing duplication at Xq28. The patient fulfilled the criteria for ASD and shared some characteristics with CK syndrome patients [16, 18, 33] but exhibited normal cognition. Further studies and case reports are required to establish that both mutations and duplications of *NSDHL* cause neurodevelopmental diseases. The relationship between *NSDHL* and ASD also requires further study to obtain conclusive support.

## Additional files

Additional file 1: **Table S1.** Results showing the Swanson Nolan and Pelham, Version IV (SNAP-IV), Autism Diagnostic Observation Schedule (ADOS) M3, Autism Diagnostic Interview-Revised (ADI-R), and Wechsler Intelligence Scale for Children (WISC) scores for the NSDHL-containing patient. **Table S2.** The DNA and cDNA primers used to analyse the NSDHL variant in the study. **Table S3–1.** The tissue expression of genes in the duplicated region of the patent in this study. **Table S3–2.** Brain expression from Allen Brian and known related diseases in the OMIM of genes in the duplicated region of the patient in this study. (DOCX 24 kb)

Additional file 2: **Figure S3.** Pyrosequencing results of mother’s genomic DNA showing X-chromosome inactivation patterns (XCIP). (DOC 31 kb)

Additional file 3: Whole exome sequencing (WES) data analysis of the patient. (DOCX 18 kb)

## Abbreviations

ADHD: Attention deficit hyperactivity disorder
ADI-R: Autism Diagnostic Review
ADOS: Autism Diagnostic Observation Schedule
ASD: Autism Spectrum Disorder
CRT: Combined Raven’s Test
CSAG1: Chondrosarcoma-associated gene
EEG: Electroencephalogram
Head MRI: Head magnetic resonance imaging
MECP2: Methyl-CpG-binding protein 2 gene
NSDHL: NAD(P)H steroid dehydrogenase-like protein
WES: Whole exome sequencing
XLID: X-linked intellectual disability
ZNF18: Zinc finger protein 185

## References

[1] Association AP (2013). Diagnostic and Statistical Manual of Mental Disorders. 5th edn (DSM-5).

[2] Lai MC, Lombardo MV, Baron-Cohen S (2014). Autism Lancet.

[3] Lichtenstein P, Carlstrom E, Rastam M, Gillberg C, Anckarsater H (2010). The genetics of autism spectrum disorders and related neuropsychiatric disorders in childhood. Am J Psychiatry.

[4] Muhle R, Trentacoste SV, Rapin I (2004). The genetics of autism. Pediatrics.

[5] Murdoch JD, State MW (2013). Recent developments in the genetics of autism spectrum disorders. Curr Opin Genet Dev.

[6] Geschwind DH (2011). Genetics of autism spectrum disorders. Trends Cogn Sci.

[7] Sanlaville D, Schluth-Bolard C, Turleau C (2009). Distal Xq duplication and functional Xq disomy. Orphanet J Rare Dis.

[8] Stankiewicz P, Thiele H, Schlicker M, Cseke-Friedrich A, Bartel-Friedrich S, Yatsenko SA (2005). Duplication of Xq26.2-q27.1, including SOX3, in a mother and daughter with short stature and dyslalia. Am J Med Genet A.

[9] Philip N, Chabrol B, Lossi AM, Cardoso C, Guerrini R, Dobyns WB (2003). Mutations in the oligophrenin-1 gene (OPHN1) cause X linked congenital cerebellar hypoplasia. J Med Genet.

[10] Van Esch H (2012). MECP2 duplication syndrome. Mol Syndromol.

[11] Samaco RC, Mandel-Brehm C, McGraw CM, Shaw CA, McGill BE, Zoghbi HY (2012). Crh and Oprm1 mediate anxiety-related behavior and social approach in a mouse model of MECP2 duplication syndrome. Nat Genet.

[12] Xu X, Xu Q, Zhang Y, Zhang X, Cheng T, Wu B (2012). A case report of Chinese brothers with inherited MECP2-containing duplication: autism and intellectual disability, but not seizures or respiratory infections. BMC Med Genet.

[13] Nakatani J, Tamada K, Hatanaka F (2009). Abnormal behavior in a chromosome-engineered mouse model for human 15q11-13 duplication seen in autism. Cell.

[14] Andersen EF, Baldwin EE, Ellingwood S, Smith R, Lamb AN (2014). Xq28 duplication overlapping the int22h-1/int22h-2 region and including RAB39B and CLIC2 in a family with intellectual and developmental disability. Am J Med Genet A.

[15] El-Hattab AW, Fang P, Jin WH, Hughes JR, Gibson JB, Patel GS (2011). Int22h-1/int22h-2-mediated Xq28 rearrangements: intellectual disability associated with duplications and in utero male lethality with deletions. J Med Genet.

[16] McLarren KW, Severson TM, du Souich C, Stockton DW, Kratz LE, Cunningham D, Hendson G, Morin RD, Wu D, Paul JE (2010). Hypomorphic temperature-sensitive alleles of NSDHL cause CK syndrome. Am J Hum Genet.

[17] Emami S, Rizzo WB, Hanley KP, Taylor JM, Goldyne ME, Williams ML (1992). Peroxisomal abnormality in fibroblasts from involved skin of CHILD syndrome. Case study and review of peroxisomal disorders in relation to skin disease. Arch Dermatol.

[18] du Souich C, Raymond FL, Grzeschik KH, Boerkoel CF. NSDHL related disorders. NCBI Bookshelf; http://www.ncbi.nlm.nih.gov/books/NBK51754/. 2011:1993–2015.

[19] Lord C, Rutter M, Lecouteur A (1994). Autism diagnostic interview-revised - a revised version of a diagnostic interview for caregivers of individuals with possible pervasive developmental disorders. J Autism Dev Disord.

[20] Lord C, Risi S, Lambrecht L, Cook EJ, Leventhal BL, DiLavore PC, Pickles A, Rutter M (2000). The autism diagnostic observation schedule-generic: a standard measure of social and communication deficits associated with the spectrum of autism. J Autism Dev Disord.

[21] Swanson JM, Kraemer HC, Hinshaw SP, Arnold LE, Conners CK, Abikoff HB (2001). Clinical relevance of the primary findings of the MTA: success rates based on severity of ADHD and ODD symptoms at the end of treatment. J Am Acad Child Psy.

[22] Wechsler D (1991). Manual for the Wechsler intelligence scale for children.

[23] Babcock RL (1994). Analysis of adult age differences on the Raven's advanced progressive matrices test. Psychol Aging.

[24] Deplaen E, Arden K, Traversari C, Gaforio JJ, Szikora JP, Desmet C (1994). Structure, chromosomal localization, and expression of 12 genes of the Mage family. Immunogenetics.

[25] Lin C, Mak S, Meitner PA, Wolf JM, Bluman EM, Block JA, Terek RM (2002). Cancer/testis antigen CSAGE is concurrently expressed with MAGE in chondrosarcoma. Gene.

[26] Chatterjee A, Tanaka T, Parrish JE, Herman GE (1995). Refined mapping of caltractin in human Xq28 and in the homologous region of the mouse X chromosome places the gene within the bare patches (Bpa) and striated (Str) critical regions. Mamm Genome.

[27] Heiss NS, Gloeckner G, Bachner D, Kioschis P, Klauck SM, Hinzmann B (1997). Genomic structure of a novel LIM domain gene (ZNF185) in Xq28 and comparisons with the orthologous murine transcript. Genomics.

[28] Sperry JB, Ryan ZC, Kumar R (2012). Hydrogen/Deuterium Exchange Reflects Binding of Human Centrin 2 to Ca(2+) and Xeroderma Pigmentosum Group C Peptide: An Example of EX1 Kinetics. Int J Mass Spectrom.

[29] Tierney E, Bukelis I, Thompson RE, Ahmed K, Aneja A, Kratz L (2006). Abnormalities of cholesterol metabolism in autism spectrum disorders. Am J Med Genet B Neuropsychiatr Genet.

[30] Cunningham D, DeBarber AE, Bir N, Binkley L, Merkens LS, Steiner RD (2015). Analysis of hedgehog signaling in cerebellar granule cell precursors in a conditional Nsdhl allele demonstrates an essential role for cholesterol in postnatal CNS development. Hum Mol Genet.

[31] Herman GE, Kratz L (2012). Disorders of sterol synthesis: beyond smith-Lemli-Opitz syndrome. Am J Med Genet C Semin Med Genet.

[32] Jakobsson M, Scholz SW, Scheet P, Gibbs JR, VanLiere JM (2008). Genotype, haplotype and copy-number variation in worldwide human populations. Nature.

[33] Firth Helen V., Richards Shola M., Bevan A. Paul, Clayton Stephen, Corpas Manuel, Rajan Diana, Vooren Steven Van, Moreau Yves, Pettett Roger M., Carter Nigel P. (2009). DECIPHER: Database of Chromosomal Imbalance and Phenotype in Humans Using Ensembl Resources. The American Journal of Human Genetics.

[34] Preiksaitiene E, Caro A, Benusiene E, Oltra S, Orellana C, Morkuniene A (2015). A novel missense mutation in the NSDHL gene identified in a Lithuanian family by targeted next-generation sequencing causes CK syndrome. Am J Med Genet A.

