# Supplementary material for: NSDHL-containing duplication at Xq28 in a male patient with autism spectrum disorder: a case report
Source: BMC Med Genet. 2018 Oct 30;19:192. doi: 10.1186/s12881-018-0705-7 (PMC6208182; doi:10.1186/s12881-018-0705-7)
Supplement: Supplementary file 1 — Table S1. Results showing the Swanson Nolan and Pelham, Version IV (SNAP-IV), Autism Diagnostic Observation Schedule (ADOS) M3, Autism Diagnostic Interview-Revised (ADI-R), and Wechsler Intelligence Scale for Children (WISC) scores for the NSDHL-containing patient. Table S2. The DNA and cDNA primers used to analyse the NSDHL variant in the study. Table S3–1. The tissue expression of genes in the duplicated region of the patent in this study. Table S3–2. Brain expression from Allen Brian and known related diseases in the OMIM of genes in the duplicated region of the patient in this study. (DOCX 24 kb) [file 12881_2018_705_MOESM1_ESM.docx]

**Table S1 Quantification of attention deficits, social interaction and cognition of the NSDHL-containing patient**

**­­­­­­­ Subject Patient**

**Swan-son Nolan and Pelham, Version IV (SNAP-IV) Aged 7years 1month**

Inattention 14/1.6

Hyperactivity/impulsivity 11/1.2

Combined type 25/1.4

Oppositional Items 17/2.1

**Autism Diagnostic Observation Schedule (ADOS) M3 Aged 8years 4 months**

Communication score (cutoff score for autism is ≥3) 4

Social interaction score (cutoff score for autism is ≥6) 6

Combined ADOS score (cutoff score for autism is ≥10) 10

Play +Stereotypic behavior and restricted interests 1+2

**Autism Diagnostic Interview-Revised (ADI-R) Aged 8years 4 months**

Qualitative Abnormalities in Reciprocal Social Interaction 16

(cutoff score=10)

Qualitative Abnormalities in Communication 12, 7

(cutoff score for verbal &nonverbal =8,7 )

Restricted Repetitive and Stereotyped Patterns of Behavior 2

(cutoff score=3)

Abnormality of Development Evident at or Before 36 Months 3

(cutoff score=1)

**Wechsler Intelligence Scale for Children (WISC) Aged 9years 8 months**

IQS of Verbal Scale 96

IQS of Performance Scales 90

Full Scale IQ 92

**Table S2 Primers used for NSDHL variant in the study**

| **Primer for** | **Primer** | **Primer sequence** |
| --- | --- | --- |
| **DNA** | Exon Forward Primer | CTTTCTCCCACTCATTCCTCAG |
|  | Exon Reverse Primer | CCCACCCTATTCCCAACTTATT |
| **cDNA** | Exon Forward Primer | CGCATGGCTGGCACATTCC |
|  | Exon Reverse Primer | ACGGTCCTCTCCATAGCATCATCC |

**Table S3-1 Tissue expression of genes in the duplicated region in Gene**

| *ZNF 185* | *NSDHL* | *MAGEA 12* | *MAGEA 6* | | *MAGEA3* | | | *MAGEA2* | *CSAG4* | *CSAG3* | *CSAG2* | *CSAG1* | *CETN2* | **tissue expression of Gene**  **(Mean PRKM)**  **Gene in the**  **duplicated region** |
| --- | --- | --- | --- | --- | --- | --- | --- | --- | --- | --- | --- | --- | --- | --- |
| 5.316±1.535 | 13.963±1.993 | - | | - | | - | - | | - | - | - | - | 30.719±3.827 | adrenal |
| 2.608±0.477 | 5.066±0.965 | - | | - | | - | - | | - | 0.079±0.068 | 0.061±0.046 | 0.076±0.08 | 15.788±2.418 | appendix |
| 2.602±0.953 | 2.772±0.441 | - | | - | | - | - | | - | - | - | - | 12.308±2.275 | bone marrow |
| **0.978±0.192** | **10.107±1.253** | **0.094±0.027** | | **-** | | **-** | **-** | | **-** | **0.054±0.064** | **0.058±0.042** | **0.168±0.039** | **28.291±1.274** | **brain** |
| 1.911±0.908 | 6.173±1.842 | - | | - | | - | - | | - | - | - | - | 15.491±2.785 | colon |
| - | 6.51±2.448 | - | | - | | - | - | | - | - | - | - | 9.666±0.212 | duodenum |
| 14.04±12.282 | 6.375±1.779 | - | | - | | - | - | | - | - | - | - | 24.998±4.936 | endometrium |
| 112.2±25.541 | 14.3380.733 | - | | - | | - | - | | - | - | - | - | 24.998±4.936 | esophagus |
| 1.518±0.134 | 11.563±2.395 | 0.082±0.043 | | - | | 0.191±0.117 | - | | - | 0.174±0.124 | 0.074±0.05 | 0.083±0.117 | 22.985±2.553 | fat |
| 3.525±0.506 | 4.689±1.02 | - | | - | | - | - | | - | - | - | - | 25.494±5.526 | gall bladder |
| - | 5.634±0.834 | - | | - | | - | - | | - | - | - | - | 16.45±2.033 | heart |
| 4.002±0.946 | 0.886±0.667 | - | | - | | - | - | | - | 0.085±0.074 | - | - | 38.151±2.829 | kidney |
| - | 9.555±1.896 | - | | - | | - | - | | - | - | - | - | 10.856±2.829 | liver |
| 5.595±0.918 | 7.142±0.907 | - | | - | | - | - | | - | 0.068±0.072 | - | - | 30.61±14.161 | lung |
| 1.679±0.254 | 4.113±0.895 | - | | - | | - | - | | - | 1.14±0.314 | 0766±0.214 | 0.231±0.136 | 10.083±1.654 | lymph node |
| - | 4.518±0.265 | - | | - | | - | - | | - | - | - | - | 33.279±0.951 | ovary |
| - | 1.083±0.109 | - | | - | | - | - | | - | - | - | - | 3.421±0.245 | pancreas |
| 11.236±2.505 | 5.095±1.406 | - | | - | | - | 0.068±0.065 | | - | - | - | - | 13.296±1.786 | placenta |
| 9.868±1.736 | 7.48±1.123 | - | | - | | - | - | | - | - | - | - | 37.352±6.814 | prostate |
| 1.689±0.344 | 2.048±0.586 | - | | - | | - | - | | - | - | - | - | 6.492±2.152 | salivary gland |
| 20.817±6.923 | 6.393±1.201 | - | | - | | - | - | | - | 0.652±0.08 | 0.427±0.089 | - | 9.601±2.085 | skin |
| - | 5.053±0.853 | - | | - | | - | - | | - | - | - | - | 11.274±1.878 | small intestine |
| 1.66±0.295 | 4.059±0.339 | - | | - | | - | - | | - | 0.811±0.448 | 0.523±0.246 | - | 10.475±0.769 | spleen |
| 3.101±1.401 | 5.089±0.638 | - | | - | | - | - | | - | - | - | - | 11.416±2.446 | stomach |
| 1.786±0.386 | 6.063±1.523 | 1.817±0.451 | | - | | 4.808±1.152 | 2.763±0.804 | | 0.261±0.131 | 1.559±0.379 | 10.25±0.233 | 0.641±0.248 | 20.494±4.775 | testis |
| 9.735±4.351 | 7.312±1.088 | - | | - | | - | - | | - | - | - | 0.070±0.094 | 47.158±15.976 | thyroid |
| 3.491±0.764 | 5.074±0.464 | - | | - | | - | - | | - | - | - | - | 23.359±3.508 | urinary bladder |

**-:tiny or no expression**

**Table S3-2 Brain expression from Allen Brian and known related diseases in the OMIM of genes in the duplicated region**

| **Genes in the**  **duplicated region** | **Brain expression from Allen Brian Atlas-Developmental Transcriptome**  **（average in log2 RPKM）** | **Known related diseases in OMIM** |
| --- | --- | --- |
| *CETN2* | 4.678 | NR |
| *CSAG1* | 0.324 | NR |
| *CSAG2* | 0.001 | N/A |
| *CSAG3* | 0.001 | N/A |
| *CSAG4* | 0.018 | N/A |
| *MAGEA2* | 0.000 | NR |
| *MAGEA3* | 0.001 | NR |
| *MAGEA6* | 0.002 | Somatic Mutation in  Pancreatic Cancer |
| *MAGEA 12* | 0.109 | NR |
| *NSDHL* | 3.710 | CHILD syndrome  CK syndrome |
| *ZNF 185* | 1.274 | downregulation may be of biologic relevance in lung carcinogenesis |

**N/A: not available NR: not been reported**
