# Supplementary material for: NSDHL-containing duplication at Xq28 in a male patient with autism spectrum disorder: a case report
Source: BMC Med Genet. 2018 Oct 30;19:192. doi: 10.1186/s12881-018-0705-7 (PMC6208182; doi:10.1186/s12881-018-0705-7)
Supplement: Supplementary file 2 — Figure S3. Pyrosequencing results of mother’s genomic DNA showing X-chromosome inactivation patterns (XCIP). (DOC 31 kb) [file 12881_2018_705_MOESM2_ESM.doc]

**Figure S3. Pyrosequencing results of the patient’s mother**
