# Supplementary material for: NSDHL-containing duplication at Xq28 in a male patient with autism spectrum disorder: a case report
Source: BMC Med Genet. 2018 Oct 30;19:192. doi: 10.1186/s12881-018-0705-7 (PMC6208182; doi:10.1186/s12881-018-0705-7)
Supplement: Supplementary file 3 — Whole exome sequencing (WES) data analysis of the patient. (DOCX 18 kb) [file 12881_2018_705_MOESM3_ESM.docx]

Whole Exome Sequencing (WES) data analysis

Item and Results

**Item:**

WES of the family trio:  the capture technologies and data analysis of WES for DNA of the patient and parents.

**Results:**

**Causative variants**: Undetectable.

**Secondary variants:** The list showed blow were the variation locus correlated with part of clinical phenotypes of the patient, but could not interpret the phenotype completely, or conform to genetic model, or have high pathogenicity, only for clinical reference.

**Table S4. List of Secondary variants identified in the patient**

| **Gene** | **Chromosome** | **mutation** | **Zygosity** | **Disease** | **Genetic mode** | **Inheritance** |
| --- | --- | --- | --- | --- | --- | --- |
| CDH15 | chr16：89260226 | NM_004933:exon13:c.2056>A(p.P686T) | Het | Mental retardation, autosomal dominant 3 [MIM:612580] | AD | Maternal |
| DIAPH3 | chr13:  6034387 | NM_001042517: c.3260-6_3260-5delT>A(p.P686T) | Het | Auditory neuropathy, autosomal dominant,1, [MIM:609129] | AD | Paternal |
| HUWE1 | chrX:53575207 | NM_031407:exon68:c.10063C>T(p.R355W) | Hemi | Mental retardation, X-linked syndromic， Turner type[MIM:300706] | X-linkd | Maternal |
| KIAA2022 | chrX:73961426 | NM_001008537:exon3:c.2966G>A(p.R989Q) | Hemi | Mental retardation, X-linked 9[MIM:300912] | X-linked | Maternal |
